# Increased cancer risk in kidney transplant patients in Scotland: a national registry linkage study

**DOI:** 10.1038/s41416-025-03086-2

**Published:** 2025-06-14

**Authors:** Ailish Nimmo, Benjamin Elyan, Joe Lakey, Stephen Marjoribanks, Shona Methven, David Morrison, Samira Bell

**Affiliations:** 1 Department of Renal Medicine, Royal Infirmary of Edinburgh, Edinburgh, UK; 2 Centre for Cardiovascular Science, University of Edinburgh, Edinburgh, UK; 3 School of Cardiovascular and Metabolic Health, University of Glasgow, Glasgow, UK; 4 Scottish Renal Registry, Public Health Scotland, Glasgow, UK; 5 Department of Renal Medicine, Aberdeen Royal Infirmary, Aberdeen, UK; 6 Scottish Cancer Registry, Public Health Scotland, Glasgow, UK; 7 Division of Population Health and Genomics, University of Dundee, Dundee, UK

**Keywords:** Cancer epidemiology, Epidemiology, Risk factors

## Abstract

**Background:**

Cancer is a major contributor to morbidity following kidney transplantation. This study examines the incidence of cancer in kidney transplant recipients (KTRs) in Scotland, compares this to the general population, and identifies factors associated with cancer development.

**Methods:**

This nationwide cohort study utilised data from the Scottish Renal Registry, Scottish Cancer Registry and hospitalisation records. Standardised incidence rate ratios (SIRs) compared cancer incidence to the general population. Cox proportional hazards models identified factors associated with post-transplant cancer.

**Results:**

Between 1997-2021, 4033 patients ≥18 years received a first kidney transplant. 770 developed cancer versus 194 expected (SIR 3.9, 95% CI 3.7–4.2). Site-specific SIRs were greatest for non-melanomatous skin cancer, lymphoma and kidney cancer. Cancer incidence was 7-times that of the general population in kidney transplant recipient (KTR) under 40, the increased incidence fell to 3-times for KTRs over 60. Consistent differences in the incidence of the most common cancers in the general population (colorectal, lung breast, prostate) were not detected in this population.

**Conclusions:**

There is an increased risk of cancer in KTRs, particularly younger individuals. Non-melanomatous skin cancers remain the most frequent cancer in a population with low natural UV exposure. Tailored counselling and surveillance strategies are needed to improve patient outcomes.

## Introduction

Kidney transplantation is the optimal treatment for many patients with end stage kidney disease (ESKD) as it is associated with improved quality and quantity of life compared to dialysis [1]. Kidney transplant recipients however experience an increased risk of cancer compared to individuals on dialysis and age and sex matched controls in the general population [2–4]. In the UK, malignancy has surpassed cardiovascular disease mortality and is the second singular leading cause of death in kidney transplant recipients [5].

The increased risk of malignancy is largely attributed to the use of immune suppression, which can alter T cell function, impair immunosurveillance, amplify patients’ susceptibility to carcinogenic agents such as UV light, and limit the control of oncogenic viral infections e.g. herpes and hepatitis viruses [6–8]. Although the incidence rates for cancer are elevated across many organ sites, the greatest increases are in viral-mediated and non-melanomatous skin cancers rather than the most frequently observed cancers in the general population [6, 8–11].

Accurate information on an individual’s risk of cancer is vital in pre-transplant counselling and can guide decisions on monitoring or screening for cancer post-transplant. With the changing demographic of the transplant population, and shifts in immune suppression regimens, there is a need to report contemporary data on cancer risk in transplant recipients [5, 12]^(p13)^. Whilst previous studies have explored cancer rates in this group, the incidence rates vary depending on geographical location, malignancy type, and comparator group [3], and many do not provide complete coverage of the transplant population, reducing the reliability and generalisability of findings.

The aims of this study were to examine the incidence of cancer in kidney transplant recipients in Scotland compared to the general population, and identify factors associated with the development of post-transplant cancer based on recipient characteristics known at the time of transplant.

## Methods

### Study population

This was an observational data linkage cohort study, comprising all patients aged ≥18 years who received a first kidney transplant between 1^st^ January 1997 and 31^st^ December 2021 in Scotland, United Kingdom.

### Data sources

The datasets utilised comprised the Scottish Renal Registry, Scottish Cancer Registry (SMR06), Scottish Morbidity Record (SMR01) and National Records for Scotland (NRS). Datasets were linked using a unique patient identifier (Community Health Index number) by the Public Health and Intelligence unit of Public Health Scotland.

The Scottish Renal Registry is a national registry of all patients receiving kidney replacement therapy (KRT) for kidney failure in Scotland, collating data from nine adult renal units covering a population of 5.44 million. It was established in 1991 and has 100% unit and patient coverage. Data held include patient demographics, KRT history, and primary renal diagnosis (PRD). The PRD groupings are described on the Scottish Renal Registry website [13]. Postcode information allows for calculation of the Scottish Government derived Scottish Index of Multiple Deprivation (SIMD) quintiles, ranging from 1 (most deprived) to 5 (least deprived) [14].

Data on the occurrence of cancer post-transplant were obtained from the Scottish Cancer Registry. This has collected population-based data on cancer since 1958, including cancer site, histology, and diagnosis date. Tumour stage and grade for certain cancers (including lung, breast, colorectal, prostate, kidney, bladder, melanoma and cervical) has been progressively introduced since 1997, with the date at which reporting started differing between cancer types [15]. Cases of cancer are identified from hospital discharge records, radiotherapy, pathology, haematology and oncology databases, cancer screening datasets, prescribing, and death records. This information creates a provisional cancer registration record, which is reviewed after six months by Cancer Information Officers who determine if the provisional registration should be confirmed [16]. Histological confirmation is not required for cancer registration; clinical diagnoses from physical examination and radiology can suffice. Outpatient records in Scotland do not have diagnosis codes and are not used to identify cases of cancer. Whole population incidence data are publicly available via Public Health Scotland, with rates calculated from mid-year population estimates [17]. The registry records the first incident cancer of each type and not recurrent or progressive disease. If a person has 2 or more cancers of different morphology or at different sites these are recorded as separate cancers.

Comorbidity at time of transplant was obtained from ICD-10 coding of any reported diagnosis from hospital admissions (daycase and inpatient) captured in SMR01 (Supplementary Table 1). Data on deaths were obtained from NRS which collects national data on all causes and dates of death that occur in Scotland using ICD-10 coding [18].

### Coding of cancers

Cancers were defined using the International Classification of Diseases, 10th Edition with codes C00-C80, as per cancer reporting for the general population (Supplementary Table 2). Only invasive cancers were included in the main analysis; data on cancers in situ are included in the Supplementary Material. The location of cancer within an organ e.g. whether kidney cancer is in a native or transplanted kidney cannot be determined. Data on the morphology of non-melanomatous skin cancers for distinction between cutaneous squamous cell and basal cell carcinomas were not available. Histological confirmation of non-melanomatous skin cancers were reported.

### Statistical methods

Baseline characteristics of continuous variables were displayed as median and interquartile range [IQR], and categorical variables summarised as percentages. Comparisons were made using the Chi-square test for categorical variables and Mann-Whitney U test for non-parametric continuous variables.

Incidence rates were calculated per 100,000 person years for the overall transplant population and for men and women separately. Duration of follow up was calculated as the time from kidney transplantation to the first incidence of cancer, death, or end of follow-up (31st December 2021). Rates of cancer were compared to the general population, and standardised incidence rate ratios (SIR) with 95% confidence intervals calculated for all cancers combined and the 8 most common cancers individually, standardised by age, sex and calendar year. SIR can be interpreted as relative risk, as it estimates the risk for transplant recipients relative to the general population, accounting for differences in age, sex, and year of diagnosis.

Univariable and multivariable Cox proportional hazards models were used to examine factors associated with the development of de-novo cancer post-transplant. Variables included were determined a priori to be non-modifiable factors known at the time of transplant with potential to associate with cancer risk, comprising age, sex, PRD, duration of dialysis pre-transplant, pre-transplant cancer (diagnosed following 1st January 1997, the start of Cancer Registry linkage), and comorbidity comprising diabetes, ischaemic heart disease, cerebrovascular disease, peripheral vascular disease, and chronic obstructive airways disease (surrogate for smoking). Data on types of pre-transplant cancer are reported. As the Cancer Registry only records the first incident cancer of each type, patients with pre-transplant cancer are only included in the de-novo cancer post-transplant group if they developed a cancer of different morphology or at a separate site. Patients were not censored at graft failure as they would still have been exposed to immune suppression, and some patients continue immune suppression after graft failure. The proportion of patients developing cancer following graft failure are reported. Era of transplantation was not included as immune suppression protocols have been broadly similar in Scotland since 2005. The proportional hazards assumption was checked using plots of Schoenfeld residuals. A sensitivity analysis was performed excluding non-melanomatous skin cancer (Supplementary Tables 3 and 4). Analyses included complete cases only, present in 3975 (98.6%) cases. Data were analysed using R (version 3.6.2, Vienna, Austria), Stata 15 (Statacorp, College Station, TX) and GraphPad Prism 8. Code can be provided upon reasonable request.

## Results

### Baseline patient characteristics

Between 1st January 1997 and 31st December 2021, 4033 patients aged ≥18 years received a first kidney transplant with a total follow up of 33,544 person years. Of these, 770 patients developed de-novo cancer post-transplant (Table 1). The median age at cancer diagnosis was 61.2 years [IQR 52.7–68.3] and the median time from transplant to cancer diagnosis 5.7 years [IQR 2.5–9.2]. There were 258 patients (6.4%) who had invasive (*n* = 187) or in situ (*n* = 71) cancer pre-transplant diagnosed after 1st January 1997. The median time between cancer diagnosis and transplantation was 5.2 years [IQR 2.7–9.1]. The most frequently observed invasive cancers prior to transplant were non-melanomatous skin cancers (*n* = 69), kidney cancer (*n* = 32), breast cancer (*n* = 13) and prostate cancer (*n* = 12). On univariate analysis, individuals who developed cancer were older, more likely to be male, have hereditary, glomerular or hypertensive causes of kidney disease, and to have been on dialysis prior to transplant rather than being transplanted pre-emptively (Table 1). Follow up time was longer for patients developing cancer (median 10.7 years vs. 6.3 years, *p* < 0.001).

**Table 1:** Baseline patient demographics.

	Incident transplant patients developing cancer	Incident transplant patients cancer free	*P* value
Number of patients	770	3263	-
Sex (male, %) (*n* = 4020)	513 (66.6)	1878 (57.8)	<0.001
Age at transplant (*n* = 4020)	54.7 (45.0–62.7)	47.7 (36.0–58.6)	<0.001
Follow up (years)	10.7 [6.5–15.0]	6.3 [3.0–11.3]	<0.001
Cause of ESKD (*n* = 4033)			
Diabetes mellitus	71 (9.2)	565 (17.3)	
Hereditary nephropathies	183 (23.8)	585 (17.9)	
Glomerular disease	254 (33.0)	891 (27.3)	
Hypertension/renovascular	37 (4.8)	150 (4.6)	<0.001
Miscellaneous renal disorders	95 (12.3)	403 (12.4)	
Other systemic diseases	9 (1.2)	50 (1.5)	
Tubulointerstitial disease	109 (14.2)	472 (14.5)	
Not coded	12 (1.6)	147 (4.5)	
First mode of KRT (*n* = 4033)			
Pre-emptive transplant	100 (13.0)	645 (19.8)	<0.001
Duration KRT pre-transplant (months)	25.7 [10.1 – 47.4]	18.8 [4.3–38.4]	<0.001
Graft status during follow-up			
Graft failure	120 (15.6)	509 (15.6)	0.99
Cancer pre-transplant Yes	52 (6.8)	206 (6.3)	0.20
SIMD (*n* = 3981)			
1	136 (17.8)	743 (23.1)	
2	171 (22.4)	703 (21.9)	
3	154 (20.2)	613 (19.1)	0.03
4	157 (20.6)	609 (18.9)	
5	146 (19.1)	549 (17.1)	
Comorbidities at transplant (*n* = 4033)			
Ischaemic heart disease	58 (7.5)	204 (6.3)	0.20
Cerebrovascular disease	20 (2.6)	145 (4.4)	0.02
Peripheral vascular disease	12 (1.6)	85 (2.6)	0.09
Diabetes mellitus	78 (10.1)	664 (20.4)	<0.001

*ESKD* end stage kidney disease, *KRT* kidney replacement therapy, *SIMD* Scottish Index of Multiple Deprivation.

Data are presented as number (%) or median [interquartile range]. *P* value reflects results from Mann-Whitney U test (continuous variables) or Chi square test (categorical variables).

### Cancer types post-transplant, incidence rates and method of detection

The most common cancers were non-melanomatous skin cancer (57.9%), lymphoma (7.9%), kidney (7.0%), lung (6.8%), breast (4.0%), and prostate (3.7%) cancer. Of non-melanomatous skin cancers, 96.9% were histologically confirmed. The number of cancers and their incidence rates per 100,000 person years are shown in Table 2. The median time to diagnosis of cancer ranged from 3-4 years for melanoma and prostate cancer to 6-7 years for lymphoma and bladder cancer.

**Table 2:** Types and incidence rate of the 10 most common de novo cancers post kidney transplantation, with median [interquartile range] time to occurance of cancer post-transplant.

Cancer type	Number of cases	Incidence rate per 100,000 person years	Median time to cancer (years)
All cancers	770	2601.5 (2423.4–2792.7)	5.7 [2.5–9.2]
Non-melanoma skin	446	1511.9 (1377.6–1659.3)	6.0 [2.8–9.1]
Lymphoma	61	207.7 (161.6–267.0)	6.9 [2.5–11.8]
Kidney	54	177.1 (134.9–232.4)	5.4 [2.5– 9.2]
Lung	53	175.4 (134.0–229.6)	5.4 [2.8–9.6]
Breast	31	105.6 (74.2–150.1)	5.0 [2.2–10.0]
Prostate	29	98.7 (68.6–142.1)	3.9 [0.8–8.6]
Colorectal	27	88.5 (60.3–130.0)	4.3 [1.8–8.3]
Bladder	20	68.1 (43.9–105.6)	6.3 [2.8–8.6]
Melanoma	17	57.9 (36.0–92.1)	3.9 [0.7–5.4]
Cervical	<5	13.6 (5.1–36.3)	4.0 [1.7–8.3]

The incidence rate and therefore absolute risk of developing cancer was greatest in the older age groups, as shown in Table 3 with a comparison to the general population.

**Table 3:** Absolute risk of developing cancer in transplant patients and the general population, based on 1997-2021 data.

Age during follow up (years)		Absolute risk in general population	Absolute risk in transplant population
All ages	Male and female	618	2602
	Female	602	2017
	Male	636	2942
18-39	Male and female	74	531
	Female	92	572
	Male	55	500
40-59	Male and female	424	2109
	Female	496	1896
	Male	349	2261
≥60	Male and female	1686	5469
	Female	1442	3716
	Male	1967	6881

Expressed as cancers per 100,000 patient-years.

Of those cancers occurring in the age ranges for which screening pathways exist, 40% of colorectal cancers (*n* = 8), 48% of breast cancers (*n* = 10) and 50% of cervical cancers (*n* < 5, exact number not shown) were detected through screening.

### Cancer incidence compared to the general population

**Fig. 1:**
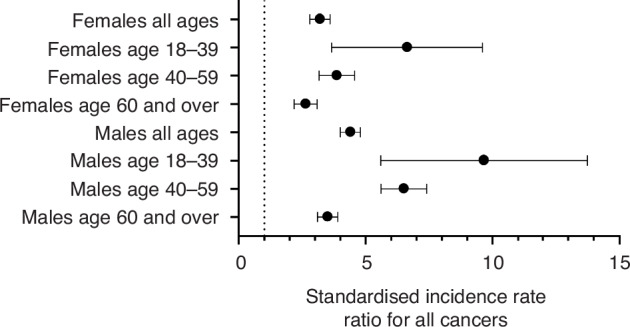
Standardised incidence rate ratio of all cancers in transplant recipients by sex and age compared to the general population.

For both sexes and across all age groups, there was an increased rate of cancer in transplant recipients compared to the general population (Table 4). The rate was greater for men and more marked for younger individuals, with the magnitude of increased rate falling with age (Fig. 1). A similar pattern was observed in a sensitivity analysis excluding non-melanomatous skin cancers (Supplementary Table 3).

**Table 4:** Risk of cancer in patients with a kidney transplant in Scotland compared to the general population.

Age during follow up (years)		Number of recipients	Years at risk	Observed cases of cancer	Expected cases of cancer	SIR (95% CI)
All ages	Male and female	4020	29955	770	194	3.9 (3.7–4.2)
	Female	1629	12689	256	80	3.2 (2.8–3.6)
	Male	2391	17266	508	115	4.4 (4.0–4.8)
18-39	Male and female	1167	7346	39	5	7.2 (5.2–9.8)
	Female	502	3160	18	3	6.2 (3.9–9.8)
	Male	705	4223	21	3	9.1 (5.9–14.0)
40-59	Male and female	2564	15268	322	65	5.0 (4.5–5.5)
	Female	1047	6380	121	32	3.8 (3.2–4.6)
	Male	1517	8888	201	31	6.5 (5.6–7.4)
≥60	Male and female	1677	7368	403	124	3.2 (29–3.6)
	Female	661	3148	117	45	2.6 (2.2–3.1)
	Male	1003	4156	286	82	3.5 (3.1–3.9)

*SIR* Standardised incidence rate ratio, calculated by indirect standardisation by age, sex and year,1997–2021.

**Fig. 2:**
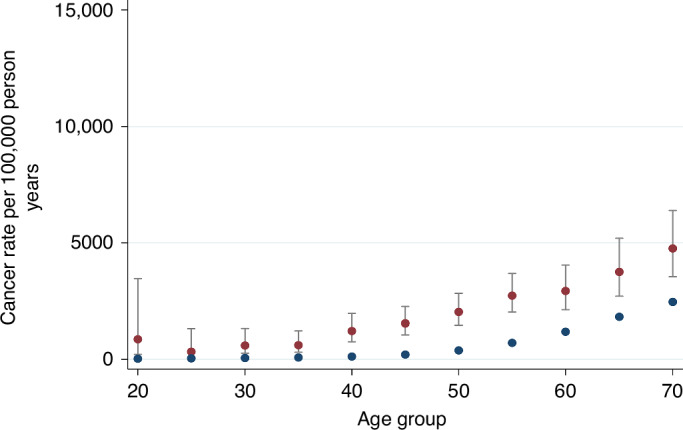
Cancer rate in female transplant recipients (red dots) versus general population (blue dots).

**Fig. 3:**
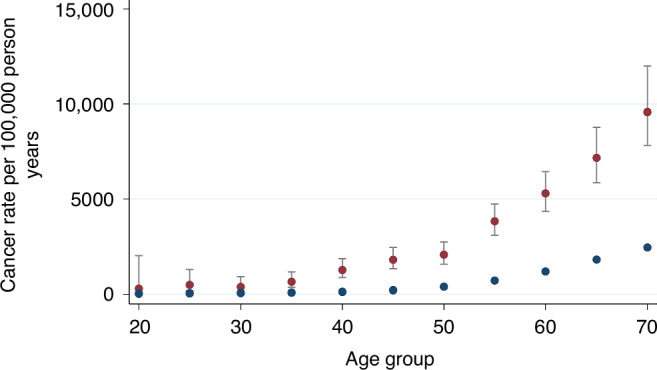
Cancer rate in male transplant recipients (red dots) versus general population (blue dots).

A female transplant recipient aged 25 had a rate of cancer equivalent to a 45-year-old woman in the general population (330 vs. 388 per 100,000 person years), and a 45-year-old female transplant recipient had a rate of cancer equivalent to a 70-year-old woman in the general population (1542 vs. 1632 per 100,000 person years) (Fig. 2). For men, a similar pattern was observed. A 30-year-old male transplant recipient had a rate of cancer equivalent to a 50-year-old man in the general population (378 vs. 387 per 100,000 person years). A 45-year-old male transplant recipient had a cancer rate equivalent to a 65-year-old man in the general population (1809 vs. 1817 per 100,000 person years) (Fig. 3).

The rate of the eight most frequent cancers in transplant recipients (Table 2) for each sex were compared to the general population. Table 5 shows the standardised incidence rate ratios of these cancers by age and sex. The rate of lymphoma was significantly higher than the general population across all age groups and both sexes (SIR 26.0 [IQR 10.8–62.5] and 14.3 [IQR 6.0–34.4] for females and males aged 18–39 respectively). In patients aged 40–59 the rate of kidney cancer was 9.4 [IQR 4.2–20.9] times higher than the general population in females and 12.2 [IQR 8.0–18.5] times higher in men. The rate of kidney cancer in those over 60 years remained elevated but to a lesser extent (SIR 3.1 [IQR 1.1–8.2] in females, SIR 5.0 [IQR 3.2–8.0] in males). The rate of colorectal cancer was greater in female transplant recipients aged 40–59 years than the general population, but this increased risk was not observed in older age groups or in men. The rate of non-melanomatous skin cancers was greater in both sexes and across all age groups, but the rate of melanoma was only increased in women aged 40–59 years. The rate of breast and prostate cancer were not increased compared to the general population. Rates of cervical cancer in situ were not different to the general population (Supplementary Material).

**Table 5:** Site-specific risk of the 9 most common solid organ cancers in kidney transplant recipients as compared to the general population, by age and sex between 1997 and 2021.

Age at cancer diagnosis (years)	Age 18-39	Age 40-59	Age ≥ 60
	SIR	SIR	SIR
Whole population			
Non-melanoma skin	13.2 (7.8 – 22.3)	10.0 (8.7 – 11.6)	6.5 (5.8 – 7.4)
Lymphoma	18.9 (10.2 – 35.2)	9.7 (6.7 – 14.0)	4.3 (2.9 – 6.5)
Kidney	21.6 (5.4 – 86.5)	12.2 (8.4 - 17.6)	4.8 (3.2 – 7.3)
Lung	-	2.5 (1.6 – 3.9)	1.1 (0.8 – 1.6)
Colorectal	-	1.9 (1.1 – 3.3)	0.63 (0.4 – 1.1)
Bladder	97.7 (24.4 – 390.5)	7.4 (3.5 – 15.5)	2.4 (1.3 – 4.3)
Melanoma	1.3 (0.2 – 9.6)	1.9 (0.9 – 4.0)	2.1 (1.1 – 4.0)
Female			
Non-melanoma skin	13.9 (6.6 – 29.1)	7.8 (5.8 – 10.1)	4.6 (3.6 – 6.0)
Lymphoma	26.0 (10.8 – 62.5)	8.9 (4.6 – 17.1)	4.8 (3.5 – 9.2)
Kidney	29.5 (4.2 – 209.4)	9.4 (4.2 – 20.9)	3.1 (1.1 – 8.2)
Lung	-	1.8 (0.7 – 4.3)	1.3 (0.8 – 2.2)
Colorectal	-	3.2 (1.6 – 6.5)	0.5 (0.2 – 1.4)
Breast	1.2 (0.2 – 8.5)	1.3 (0.9 – 2.1)	0.7 (0.4 – 1.3)
Bladder	-	8.0 (2.0 – 32.1)	3.0 (1.0 – 9.4)
Melanoma	2.3 (0.3 – 16.6)	1.8 (1.6 – 5.5)	2.8 (1.0 – 7.4)
Male			
Non-melaoma skin	13.0 (6.2 – 27.3)	11.3 (9.4 – 13.5)	6.9 (6.0 – 7.9)
Lymphoma	14.3 (6.0 – 34.4)	9.7 (6.2 – 15.1)	3.9 (2.3 – 6.6)
Kidney	16.5 (2.3 – 116.8)	12.2 (8.0 – 18.5)	5.0 (3.2 – 8.0)
Lung	-	2.8 (1.6 – 4.9)	1.0 (0.6 – 1.5)
Colorectal	-	1.1 (0.5 – 2.7)	0.6 (0.3 – 1.2)
Prostate	-	1.7 (0.8 – 3.5)	0.8 (0.6 – 1.3)
Bladder	155.7 (38.9 – 622.4)	6.5 (2.7 – 3.8)	1.9 (0.9 – 3.8)
Melanoma	-	2.2 (0.8 – 5.8)	1.7 (0.7 – 4.0)

*SIR* standardised incidence rate ratio. SIRs marked as ‘-‘ refer to cancers where no cases were observed.

### Cancer after graft failure

Over follow up, 629 (15.6%) patients experienced graft failure and returned to dialysis or were re-transplanted, with a median time to graft failure of 4.1 years [IQR 0.8-6.4]. Of the 770 patients who developed cancer, 710 (92.2%) developed cancer prior to failure of their first transplant. Of non-melanomatous skin cancers (*n* = 446), 421 (94.4%) were diagnosed during the lifespan of the first transplant, 17 (3.8%) during a subsequent transplant, and 8 (1.8%) following graft failure whilst on dialysis. Of lymphomas (*n* = 61), 58 (95.1%) were diagnosed during the lifespan of the first transplant, of kidney cancers (*n* = 54), 45 (83.3%) were diagnosed during the lifespan of the first transplant, and of breast cancers (*n* = 31), 25 (80.7%) were diagnosed during the lifespan of the first transplant. All cases of lung cancer (*n* = 53) were diagnosed during the lifespan of the first functioning transplant.

### Factors associated with the development of cancer

On univariable Cox regression (Table 6), individuals developing cancer were older (HR 1.06, 95% CI 1.07–1.07), more likely to be male (HR 1.29, 95% CI 1.13–1.48), and more likely to have glomerular (HR 1.66, 95% CI 1.28–2.17), familial (HR 1.80, 95% CI 1.37–2.38) or hypertensive (HR 1.71, 95% CI 1.15–2.55) PRDs than diabetes. A trend towards an increased cancer risk in those from higher socio-economic classes was observed. A longer duration of KRT pre-transplant and a prior history of cancer also associated with post-transplant cancer.

**Table 6:** Cox model examining risk factors for cancer in kidney transplant recipients.

		Model	
		Unadjusted	Adjusted
Age (per year)	HR (95% CI) *P*	1.06 (1.05–1.07) **<0.001**	1.06 (1.05–1.07) **<0.001**
Female sex	HR (95% CI) *P*	0.67 (0.58–0.79) **<0.001**	0.63 (0.54-0.74) **<0.001**
PRD: Ref DM			
Familial	HR (95% CI)	1.80 (1.37–2.38)	1.14 (0.76– 1.72)
	*P*	**<0.001**	0.53
Glomerular	HR (95% CI)	1.66 (1.28–2.17)	1.21 (0.82–1.80)
	*P*	**<0.001**	0.34
Hypertension	HR (95% CI)	1.71 (1.15–2.55)	0.79 (0.48–1.30)
	*P*	**0.008**	0.34
Miscellaneous	HR (95% CI)	1.28 (0.94–1.74)	0.91 (0.59–1.41)
	*P*	0.12	0.68
Systemic	HR (95% CI)	1.33 (0.64–2.78)	1.06 (0.48–2.34)
	*P*	0.44	0.89
Tubulointerstitial	HR (95% CI)	1.24 (0.92–1.68)	1.06 (0.70–1.61)
	*P*	0.16	0.79
Not coded	HR (95% CI)	0.57 (0.31–1.06)	0.59 (0.30–1.14)
	*P*	0.07	0.12
SIMD: Ref SIMD1			
SIMD 2	HR (95% CI)	1.21 (0.97–1.52)	1.20 (0.96–1.50)
	*P*	0.10	0.12
SIMD 3	HR (95% CI)	1.29 (1.02–1.62)	1.20 (0.95–1.51)
	*P*	**0.03**	0.13
SIMD 4	HR (95% CI)	1.27 (1.01– 1.60)	1.13 (0.90–1.42)
	*P*	**0.04**	0.30
SIMD 5	HR (95% CI)	1.38 (1.10–1.75)	1.24 (0.98–1.57)
	*P*	**0.006**	0.08
Pre-transplant KRT (per year)	HR (95% CI) *P*	1.11 (1.08–1.14) **<0.001**	1.03 (1.00–1.07) **0.04**
Cancer pre-transplant	HR (95% CI) *P*	1.93 (1.45–2.57) **<0.001**	1.33 (1.00– 1.78) **0.05**
History diabetes	HR (95% CI) *P*	0.70 (0.55–0.89) **0.003**	0.74 (0.51–1.07) 0.11
History IHD	HR (95% CI) *P*	1.80 (1.37–2.36) **<0.001**	1.07 (0.81–1.42) 0.62
History CVD	HR (95% CI) *P*	0.91 (0.57–1.43) 0.67	0.67 (0.42–1.08) 0.10
History PVD	HR (95% CI) *P*	0.85 (0.48 –1.50) 0.58	0.95 (0.53–1.72) 0.87
History COPD	HR (95% CI) *P*	1.49 (0.84–2.64) 0.17	0.90 (0.51–1.61) 0.73

*PRD* primary renal diagnosis; *DM* diabetes mellitus; *SIMD* Scottish Index of Multiple Deprivation; *KRT* kidney replacement therapy; *IHD* ischaemic heart disease; *CVD* cerebrovascular disease; *PVD* peripheral vascular disease; *COPD* chronic obstructive pulmonary disease.

On multivariable analysis, factors that remained associated with cancer development comprised increased age (aHR 1.06, 95% CI 1.05-1.07), male sex (aHR 1.39, 95% CI 1.21-1.60), a longer duration of dialysis pre-transplant (aHR 1.03, 95% CI 1.00–1.07) and history of cancer pre-transplant (aHR 1.33, 95% CI 1.00–1.78).

### Cancer stage

Cancer stage at diagnosis for transplant recipients and the general population is shown in Supplementary Table 5. Cancer stage was similar between groups, aside from bladder cancer where a greater proportion of transplant recipients presented with stage 4 disease.

### Cause of death

Over follow up, 981 patients died. Of the 770 patients who developed cancer post-transplant, 348 patients died. In the total cohort, malignancy accounted for 186 (19.0%) of deaths, the third most common cause of death behind other causes (*n* = 354, 36.1%; includes kidney failure) and cardiovascular disease (*n* = 275, 28.0%). There were 166 deaths attributed to infection (16.9%), of which 73 (44.0% of infective deaths) related to COVID-19. In those patients with a history of post-transplant cancer, 176 deaths were attributed to malignancy (50.6%). The next most frequent causes of death were other causes (*n* = 73, 20.1%) and cardiovascular disease (*n* = 59, 17.0%). The 10 deaths related to cancer from NRS that were not identified in the Cancer Registry may relate to the cancer being diagnosed outwith Scotland or the Registry determining based on further information that the patient did not have cancer.

## Discussion

This study describes the epidemiology of cancer in 4033 incident kidney transplant recipients in Scotland, followed up for a median of over 6 years. Compared with the general population, there is an increased risk of cancer, in particular non-melanomatous skin cancer, lymphoma and kidney cancer. The increased risk was greatest in transplant recipients under 40 years, at 7 times that of the general population, whilst recipients aged 60 years and over had a cancer rate 3 times that of the general population. The incidence of the most common cancers in the general population, including lung, breast, colorectal, and prostate, were minimally or not increased post-transplant in this study.

Our findings align with previous studies identifying risk factors for post-transplant cancer, including older age at transplantation, male sex, longer duration of dialysis pre-transplant, and prior history of cancer. In 6000 transplant recipients in Australia and New Zealand, pre-transplant dialysis duration was an independent risk factor for cancers of the urinary tract and lung, highlighting a need for vigilance in individuals with greater dialysis vintage [19]. Similarly, a US study of over 200,000 transplant recipients reported skin cancer risk was associated with older age, male sex, White ethnicity and pre-transplant malignancy [20]. Exposure to UV radiation and susceptibility of keratinocytes are key factors in the development of non-melanomatous skin cancer, and likely explains the variation in risk by geographical location with skin cancers particularly prevalent in Australia and New Zealand where there is high UV exposure [21, 22]. It is important to note however that even in Scotland, where vitamin D deficiency due to lack of UV exposure is common [23], non-melanomatous skin cancers still comprised over 50% of all cancers. Individuals with a history of cancer are also at an increased risk of recurrence. A study from Italy found nearly 40% of patients with non-melanomatous skin cancer developed a second lesion, and patients with a primary non-cutaneous cancer had an increased risk of developing a second non-cutaneous cancer [24].

Previous studies have identified further risk factors for post-transplant cancer. In 7000 patients in Australia and New Zealand, recipients of extended criteria deceased donor kidneys had a 1.5 fold increased risk of cancer, particularly genitourinary cancer and lymphoma, compared to those receiving a live donor organ [25]. Immune suppression also impacts on cancer risk, with pre-transplant cyclophosphamide or rituximab for the treatment of glomerular diseases [26], and the use of lymphocyte depleting agents being identified as risk factors [27, 28]. Cancers with a viral aetiology are more frequently observed during periods with a functioning graft when immune suppression is greatest, whilst CKD-related cancers such as kidney cancers may have a higher incidence after transplant failure [29]. The role of viral infections in the aetiology of post-transplant cancer has been most clearly defined with Epstein-Barr virus, with recipient serostatus a major determinant of lymphoma risk [30]. BK virus may also be implicated in urothelial malignancy, with studies showing an increased rate of bladder cancer in patients with BK nephropathy or BK viraemia [31], and a fifth of bladder cancers in transplant recipients containing BK viral sequences [32].

Cancer-related mortality is greater in transplant recipients than the general population [33]. The stage of cancer at presentation is a key factor in outcomes but we found cancer stage at diagnosis similar to the general population, aside from bladder cancer which tended to present more advanced. This differs from a study of 635 transplant recipients from the Israel Penn registry, which found that aside from kidney cancer most de novo cancers were more advanced at presentation than the general population [34]. A study of over 2500 transplant recipients in Australia and New Zealand with de novo cancer found the proportion presenting with lymph node or metastatic spread was approximately 20% for breast, 45% for lung, and 40% for colorectal cancer [35], similar to what is reported here. The high proportion of cases of advanced bladder cancer at presentation here merits discussion. As all transplant patients are informally screened with urinalysis at every clinic visit, it is a concern bladder cancer is not diagnosed early. This may reflect complacency regarding non-visible haematuria, which could also represent recurrent renal disease, or the disease being more aggressive in transplant recipients.

International guidelines recommend transplant recipients undergo the same solid organ cancer screening as the general population, though different diagnostic sensitivities, increased competing risks for deaths e.g. from cardiovascular disease, and reduced life expectancy in transplant recipients may impact screening effectiveness [36–38]. In Scotland, national screening programmes exist for cervical (cervical smear every 5 years for women aged 25–64 years), breast (mammogram every 3 years for women aged 50–70 years), and colorectal cancer (faecal occult blood or faecal immunochemical testing every 2 years between 50–74 years) [39]. The proportion of cancer cases picked up by screening in eligible transplant recipients was similar to that in the general population (around 30% of colorectal, 50% of breast and 40% of cervical cancers), although data on the uptake of screening among transplant patients compared to the general population was not available [15]. Screening for kidney cancer is not currently recommended, with a Markov model suggesting minimal survival benefit and an unfavourable cost-effectiveness ratio [40] which may relate to poor sensitivity of ultrasound in detecting lesions in abnormal end-stage kidneys [38]. The increased rate of kidney cancers in our cohort should however be noted, and a high index of suspicion should be maintained by transplant physicians. For skin cancers, guidance is for regular self-examination, and for a skin review biannually for the first 5 years post-transplant and annually thereafter [37]. A 2020 UK survey however found only 55% of transplant centres have a skin surveillance service, and of those that do two-thirds of assessments were performed by a non-skin cancer specialist [41]. Ensuring appropriate funding and training is available to facilitate such services and enable collaboration between skin cancer and transplant specialists is key to improving skin surveillance rates, which is vital given the high incidence rate of skin cancers in our population.

Whilst the increased risk of cancer post-transplant can be explained by immune suppression and susceptibility to viral-mediated cancers, ascertainment bias should also be considered. Kidney transplant recipients are closely monitored and frequently experience symptoms such as gastrointestinal disturbance relating to transplant medications. Such symptoms might prompt cross-sectional imaging and could explain the increased rate of urinary tract cancers, though no consistent increased rate of lung or colorectal cancer were seen in our population. Similarly, regular skin examinations provide opportunity for diagnosing skin cancers that may not have been detected until later in the disease course, when competing risks such as cardiovascular disease may have meant they would not have had a clinical impact on the patient.

This study has several strengths. We provide contemporary data with complete geographical coverage and a long duration of follow up for the population of Scotland, where there is universal access to health care. Results are generalisable to other high-income countries and provide vital information for patients and clinicians involved in transplant care. The examined registries have high accuracy and completeness rates [42], allow examination of cancer staging in transplant recipients and the general population, and enable comparison to other transplant populations across the world.

There are limitations to this study. Firstly, data on immune suppression were not available. Kidney transplantation in Scotland is performed in 2 centres with similar practice. Standard immune suppression prior to 2000 comprised methylprednisolone induction with prednisolone, ciclosporin and azathioprine maintenance. Basiliximab induction was introduced around 2000, and maintenance immune suppression changed to prednisolone, tacrolimus and mycophenolate mofetil between 2005–2007. Secondly, this study has not accounted for how time post-transplant impacts on the risk of cancer. This is likely to be non-linear i.e. there may be a cumulative risk relating to time on immune suppression that results in a greater risk with increasing time post-transplant, coupled with the lag time between exposure, cancer development and subsequent detection. Data were not available on other potential risk factors for cancer, such as smoking status, ethnicity, body mass index, sun exposure, donor type or viral mismatches. It is possible that some cancers, in particular non-melanomatous skin cancers, are under-reported in the Cancer Registry. This is because some may be treated in primary care without further records of the diagnosis, and others may not be brought to medical attention. Cancers however do not require pathology to be registered e.g. skin cancers treated with cryotherapy, and under-ascertainment of non-melanomatous skin cancers should not differentially affect transplant patients or lead to artefactual differences in apparent incidence compared to the general population. Finally, our data span the era of COVID-19 which may impact on incidence rates, as observed in the general population [43].

In conclusion, this study shows the rate of cancer in kidney transplant recipients is equivalent to someone 20 years older in the general population. The most frequently observed cancers were non-melanomatous skin cancers, lymphoma, and kidney cancer, reflecting a different distribution to the general population. The risk of cancer differs with age, sex, prior duration of dialysis, and pre-transplant malignancy, with younger transplant recipients having a disproportionate increased relative risk of cancer. These findings can guide patient counselling prior to transplantation, emphasise the importance of engaging transplant recipients in cancer surveillance, and highlight the need for robust pathways for diagnosing and treating cancer in kidney transplant recipients.

## Supplementary information

Supplementary Material

## Data Availability

The data underlying this article are available in the article and in its online supplementary material.
