# Supplementary material for: Increased cancer risk in kidney transplant patients in Scotland: a national registry linkage study
Source: Br J Cancer. 2025 Jun 14;133(4):555–63. doi: 10.1038/s41416-025-03086-2 (PMC12356882; doi:10.1038/s41416-025-03086-2)
Supplement: Supplementary file 1 — Supplementary Material [file 41416_2025_3086_MOESM1_ESM.docx]

**Supplementary Material**

| Comorbidity | ICD-10 Code |
| --- | --- |
| Ischaemic heart disease | I20 - I25 |
| Cerebrovascular disease | I60-I69, G45-G46, S065-S066 |
| Peripheral vascular disease | I739 |
| Diabetes mellitus | E10-E14 |
| Chronic obstructive airways disease | J44 |

**Supplementary Table 1. ICD-10 codes used for comorbidity identification.**

| Cancer type | ICD-10 Code |
| --- | --- |
| All cancers | C00 – C97 |
| Breast | C50 |
| Bladder | C67 |
| Colorectal | C18-20 |
| Kidney | C64-65 |
| Lung (trachea, bronchus and lung) | C33-34 |
| Lymphoma (Hodgkin and non-Hodgkin) | C81-86 |
| Melanoma | C43 |
| Non-melanomatous skin cancers | C44 |
| Prostate | C61 |
| Carcinoma in situ of breast | D05 |
| Cervical | C53 |
| Carcinoma in situ of cervix | D06 |

**Supplementary Table 2. ICD-10 codes used for detecting cancers as per those used by the Scottish Cancer Registry reporting for the general population.**

| Age during follow up (years) | | Number of recipients | Years at risk | Observed cases of cancer | Expected cases of cancer | SIR (95% CI) |
| --- | --- | --- | --- | --- | --- | --- |
| All ages | Male and female | 4020 | 32577 | 320 | 162 | 2.0 (1.8 – 2.2) |
|  | Female | 1629 | 13431 | 139 | 67 | 2.1 (1.8 – 2.5) |
|  | Male | 2391 | 19081 | 181 | 95 | 1.91 (1.7 – 2.2) |
| 18-39 | Male and female | 1167 | 7401 | 25 | 4 | 5.6 (3.8 – 8.4) |
|  | Female | 502 | 3174 | 11 | 2 | 4.5 (2.5 – 8.2) |
|  | Male | 705 | 4227 | 14 | 2 | 7.8 (4.6 – 13.2) |
| 40-59 | Male and female | 2564 | 16085 | 148 | 51 | 2.9 (2.5 – 3.4) |
|  | Female | 1047 | 6655 | 69 | 26 | 2.6 (2.1 – 3.4) |
|  | Male | 1517 | 9430 | 79 | 22 | 3.6 (2.9- 4.5) |
| >60 | Male and female | 1677 | 9091 | 147 | 107 | 1.4 (1.2 – 1.6) |
|  | Female | 661 | 3602 | 59 | 38 | 1.5 (1.2 – 2.0) |
|  | Male | 1003 | 5424 | 88 | 71 | 1.2 (1.0 – 1.50 |

**Supplementary Table 3. Risk of cancer in patients with a kidney transplant in Scotland compared to the general population, excluding non-melanomatous skin cancer. SIR: Standardised incidence rate ratio, calculated by indirect standardisation by age, sex and year,1997-2021.**

|  |  | Model | |
| --- | --- | --- | --- |
|  |  | **Unadjusted** | **Adjusted** |
| Age (years) | HR (95% CI)  *P* | 1.03 (1.02 – 1.04)  **<0.001** | 1.03 (1.02 – 1.04)  **<0.001** |
| Female sex | HR (95% CI)  *P* | 1.11 (0.89 – 1.39)  0.35 | 1.11 (0.89 – 1.40)  0.35 |
| PRD: Ref DM  Familial  Glomerular  Hypertension  Miscellaneous    Systemic  Tubulointerstitial  Not coded | HR (95% CI)  *P*  HR (95% CI)  *P*  HR (95% CI)  *P*  HR (95% CI)  *P*  HR (95% CI)  *P*  HR (95% CI)  *P*  HR (95% CI)  *P* | 1.28 (0.84 – 1.96)  0.25  1.23 (0.82 – 1.84)  0.32  1.95 (1.11 – 3.40)  **0.02**  1.17 (0.72 – 1.86)  0.52  2.13 (0.89 – 5.10)  0.09  1.62 (1.06 – 2.49)  **0.03**  0.43 (0.15 – 1.22)  0.11 | 1.43 (0.78 – 2.62)  0.24  1.55 (0.87 – 2.78)  0.14  1.84 (0.91 – 3.73)  0.09  1.37 (0.73 – 2.59)  0.33  2.95 (1.11 – 7.88)  **0.03**  2.05 (1.13 – 3.70)  **0.02**  0.64 (0.21 – 1.90)  0.42 |
| SIMD: Ref SIMD1  SIMD 2  SIMD 3  SIMD 4  SIMD 5 | HR (95% CI)  *P*  HR (95% CI)  *P*  HR (95% CI)  *P*  HR (95% CI)  *P* | 0.94 (0.69 – 1.29)  0.72  0.70 (0.49 – 1.00)  0.05  0.81 (0.58 – 1.14)  0.23  0.77 (0.54 – 1.10)  0.16 | 0.93 (0.68 – 1.27)  0.65  0.64 (0.45 – 0.91)  **0.02**  0.75 (0.53 – 1.06)  0.10  0.73 (0.51 – 1.04)  0.08 |
| Duration KRT pre-transplant | HR (95% CI)  *P* | 1.00 (1.00 – 1.01)  **<0.001** | 1.00 (1.00-1.00)  **0.005** |
| Cancer pre-transplant | HR (95% CI)  *P* | 1.34 (0.82 - 2.19)  0.24 | 0.94 (0.57 – 1.55)  0.81 |
| History diabetes | HR (95% CI)  *P* | 0.96 (0.69 – 1.34)  0.82 | 1.36 (0.83 – 2.24)  0.22 |
| History IHD | HR (95% CI)  *P* | 0.80 (0.46 – 1.39)  0.42 | 0.52 (0.29 – 0.90)  **0.02** |
| History CVD | HR (95% CI)  *P* | 1.12 (0.60 – 2.11)  0.72 | 1.02 (0.54 – 1.94)  0.94 |
| History PVD | HR (95% CI)  *P* | 1.20 (0.57 – 2.550  0.63 | 1.08 (0.50 – 2.34)  0.86 |

**Supplementary Table 4. Cox model examining risk factors for cancer in kidney transplant recipients, excluding non-melanomatous skin cancers.**

**Abbreviations: PRD primary renal diagnosis; DM diabetes mellitus; SIMD Scottish Index of Multiple Deprivation; KRT kidney replacement therapy; IHD ischaemic heart disease; CVD cerebrovascular disease; PVD peripheral vascular diease.**

| Cancer | Patient group | Percentage presenting at each cancer stage | | | | |
| --- | --- | --- | --- | --- | --- | --- |
|  |  | **1** | **2** | **3** | **4** | **Unknown** |
| Colorectal | Transplant recipients (n=12) | 41.7 | 0 | 8.3 | 25.0 | 25.0 |
|  | General population | 15.1 | 22.6 | 23.7 | 29.1 | 18.5 |
| Kidney | Transplant recipients (n=40) | 42.5 | 2.5 | 17.5 | 20.0 | 17.5 |
|  | General population | 41.5 | 4.9 | 20.1 | 22.1 | 11.4 |
| Lung | Transplant recipients (n=52) | 23.1 | 3.9 | 17.3 | 44.2 | 11.5 |
|  | General population | 20.3 | 6.9 | 20.2 | 46.1 | 6.4 |
| Breast | Transplant recipients (n=31) | 38.7 | 32.3 | 12.9 | 9.7 | 6.5 |
|  | General population | 40.2 | 38.1 | 9.6 | 6.3 | 5.9 |
| Prostate | Transplant recipients (n=23) | 0 | 17.4 | 21.7 | 26.1 | 34.8 |
|  | General population | 11.6 | 15.8 | 24.1 | 26.9 | 21.5 |
| Bladder | Transplant recipients (n=11) | 9.1 | 27.3 | 0 | 27.7 | 36.4 |
|  | General population | 21.0 | 18.0 | 11.7 | 11.5 | 37.9 |
| Melanoma | Transplant recipients (n=6) | 50.0 | 16.7 | 0 | 33.3 | 0 |
|  | General population | 29.2 | 14.6 | 7.3 | 2.7 | 46.2 |

**Supplementary Table 5. Cancer stage at diagnosis in transplant recipients and the general population, for cancers where this data is routinely reported. Note that the dates in which cancer stage reporting commenced differs between cancer types, and only transplant recipients with a diagnosis within these timeframes are included in this table. General population data is from reporting in 2021.**

**Supplementary data on patients with cancer in situ**

There were an additional 304 cases of cancer in situ over follow up, comprising 270 cases of non-melanomatous skin cancer, 17 cases of cervical cancer in situ, 10 cases of melanoma in situ, and <5 cases of colorectal cancer in situ and breast cancer in situ. Of those for which screening programmes exist, 94% of the cervical cases, 67% of the breast cases and 25% of the colorectal cases were identified through screening. Population data on cancer incidence is only available for cervical carcinomas in situ. The rate of cervical cancer in situ was not different between kidney transplant recipients and the general population (age 18-39 SIR 1.29, 95% CI 0.73 - 2.28, age 40-60 SIR 1.34, 95% CI 0.56 – 3.21).
